# Intimate partner violence among reproductive-age women in central Gondar zone, Northwest, Ethiopia: a population-based study

**DOI:** 10.1186/s12905-022-01685-2

**Published:** 2022-04-09

**Authors:** Wondale Getinet, Telake Azale, Eskedar Getie, Endalamaw Salelaw, Tadele Amare, Demeke Demilew, Alemu Lemma, Destaw Kibret, Abayneh Aklilu, Techalo Tensae, Mengesha Srahbzu, Shegaye Shumet

**Affiliations:** 1 Department of Psychiatry College of Medicine and Health Science, University of Gondar, Gondar, Ethiopia; 2 Institute of Public Health College of Medicine and Health Science, University of Gondar, Gondar, Ethiopia; 3 School of Midwifery College of Medicine and Health Science, University of Gondar, Gondar, Ethiopia; 4 Central Gondar Zonal Health Office, Gondar, Ethiopia

**Keywords:** Women, Violence, Intimate partner violence, Determinant factors, Ethiopia

## Abstract

**Background:**

Violence against women is the most widespread kind of human rights violation, and it has been linked to a wide range of consequences. The most prominent psychosocial and mental health concern that has serious effects for women's physical and mental well-being. This study assessed the prevalence and associated factors of women’s violence by intimate partner among women in the reproductive age group (15–49).

**Methods:**

Multistage community-based cross-sectional study was conducted among reproductive age group women in the central Gondar zone. We recruited 845 participants and interviewed by health extension workers using face-to-face interviews. We used a Women's Abuse Screening test to outcome variable; it has a total score ranges 0–16, a score > 1 indicates positive for the presence of intimate partner violence within a year. Variables were coded and entered to Epi data version 3.1 and exported to SPSS version 21 for analysis. Descriptive statistics and multivariate logistic regression analysis was run for data analysis. Adjusted odds ratios (AOR) with a 95% confidence level (CI) were declared significant.

**Result:**

Among a total of 845 participants 804 responded to the interviews with a response rate of 95%. The prevalence rate of intimate partner violence is 391(48.6%). From multivariate logistic regression analysis women being married [AOR:3.85; 95% CI (2.38, 6.22)], high school and above educational status [(AOR: 0.43; 95% CI (0.30, 0.61), women’s having > 3children [(AOR: 1.82, 95% CI (1.0, 3.1)], having a household food insecurity[(AOR: 2.09, 95% CI (1.51, 2.91)], having life threatening events [(AOR: 2.09; 95% CI (1.51, 2.91)], moderate social support [(AOR: 0.60; 95% CI (0.41, 0.83)], depression [(AOR: 3.12; 95% CI (1.60, 6.07) were significantly associated with violence by intimate partner at 95% CI .

**Conclusion:**

Intimate partner violence is common among reproductive-age women. Married, women with several children, food insecurity, life-threatening events, and depression were all found to be significant predictors of violence. Measures should be taken to raise community awareness, particularly among intimate partners, their families, and government officials.

## Introduction

Having gender-based violence (GBV) is taken into account as a psychosocial and women rights issue [1]. From types of GBV violence by intimate partner (IPV) is the first one. An intimate partner violence is a behavior within an intimate relationship that causes physical, psychological, or sexual harm in intimates [2, 3]. Both females and males are risk to partner violence, but evidence shows high prevalent in women [4]. Violence on women by their intimate partner is one of a major public health problem and a violation of women's rights [2, 3, 5–11]. World health organization (WHO) report shows 30% of women worldwide have been subjected to either physical and/or sexual partner violence globally [4]. Then, almost one-third of reproductive age women aged (15–49) years who have been sexually active and in a relationship have been exposed to violence by their intimate partner [12].

Violence is common human behavior irrespective of ethnic/racial group, but the grade and magnitude that is tolerable may differ from one society to another society [13]. From global perspective, Sub-Saharan countries accounts large number of partner violence against women with a total of 36% outnumber the global average of 30% [3].

Intimate partner violence had multiple adverse effects on women physical, mental, sexual, and reproductive health that have both short and long-term effects like miscarriage and sexually transmitted infections, unintended pregnancies, induced abortions, gynecological problems, depressive disorder, stress and other anxiety disorders, sleep difficulties, eating disorders, homicide, or suicide [12, 14–16]. Severe consequences for women physical and mental well-being [17, 18].

A study conducted among women showed that 10% to 58% have experienced physical violence in their lifetimes [19], in Ethiopia a one year and lifetime physical violence was 26% and 31% [20]. After implementation of locked down at home travel ban due to covid-19 pandemic domestic violence was increased in different countries like Australia two-thirds of women experienced physical or sexual violence by a current or former partner [21], domestic violence increased three times in China after imposing quarantine [22], America 21–35% increase domestic violence [23].

Several risk factors exacerbating violence against women that have relation to intimate partner violence perpetration. They might not be direct causes but, may contributing such as, low income, low academic achievement, alcohol use, depression, unemployment, food insecurity [24–26]. A mixture of varied personal, community, and societal factors contribute to possibility of becoming evidence for women’s violence [26]. Therefore, as a solution knowing different contributing factors may help in identifying multiple opportunities for prevention strategies. Regardless of the increasing magnitude of violence worldwide, there is little evidence on violence against women of reproductive age. Therefore, this research aimed in identifying prevalence of intimate partner violence and associated factors among reproductive-age women in Northwest Ethiopia.

## Methods

### Study design, area, population

A multistage community based cross-sectional study design employed to address parts of the study objectives. It was conducted in central Gondar zone, Amhara, Ethiopia. One of the largest and densely populated regions, Bahir Dar town is the regional city which is around 560 km away from the capital city of Ethiopia. Seven years ago, report published by the Central Statistical Agency in 2007 Ethiopia, Amhara region has a total 20million people living in twelve zones. A multistage Community based cross-sectional study conducted among reproductive age women in, Northwest Ethiopia, from October 2020 to May 2021. All reproductive age women who were residing in study area and who were available during data collection period are source populations. All methods were performed in accordance with the relevant guidelines and regulations**.**

### Sample size and sampling procedure

The sample size was calculated using a single population proportion formula that considered the following assumptions: We used a 50% prevalence of intimate partner violence, 95% confidence level, and a 5% margin of error because we were unable to get a previous study among reproductive-age women in Ethiopia (absolute level of precision). The current study used a multistage cluster sampling strategy to enroll study participants. The design effect accounts for the possible presence of inter-cluster variability in the multi-stage sampling approach. In this case, the design effect is supposed to be comparable to the number of phases that were passed through to reach the final respondents. However, because of limited resources to conduct the study, we used our design effect to 2. Additionally, a non-response rate of 10% considered and eventually, a sample size of 845 calculated. Within the process of attending to the individual study participant, a lottery method was employed to pick out three woredas randomly. Then 15 kebeles (5 kebeles in each of the randomly selected woredas) were selected from the randomly selected words (Gondar Zuria, East Denbiya, and Wogera). Finally, reproductive age women (15–49) included. All households with reproductive-age women who have intimate partner were included and interviewed in clusters. This made the final number of respondents 845. The first house was selected randomly by the lottery method. Whenever over one eligible reproductive age woman was found in the selected household, only one invited to participate by lottery method for interview. But no eligible reproductive age women in selected house, or the chosen house is closed in another revisit, we continue to the subsequent house until getting an eligible woman.

$${\text{A}}\,{\text{formula}}:\quad {\text{n }} = \left( {{\text{Z}}\alpha /{2}} \right){2 }*{\text{ P }}\left( {{1} - {\text{P}}} \right)/{\text{ d2}}\;{\text{use}}\,{\text{for}}\,{\text{sample}}\,{\text{calculation}}$$

We got, $${\text{n}} = \frac{{\left( {1.96} \right)2\left[ {0.5*\left( {1 - 0.5} \right)} \right]}}{{\left( {0.05} \right)2}} = 384.$$

By multiplying with design effect 2 and including10% non-response rate makes the final sample size 845.

### Instrument for data collection

A structured questionnaire was used to collect data during a face-to-face interview at the participants' homes. The questionnaires were written in English and then translated into Amharic, the local language of the catchment region. The data collection technique involved fifteen health extension workers and three supervisors. Local guiders also help to choose suitable women for each home. Designing data collection materials was given a lot of thought to ensure the quality of the data collected. For five days, data collectors and supervisors received data collection training. Data collectors, supervisors, and the investigator evaluated questionnaires for completeness daily during data collection.

#### Intimate partner violence (IPV)

Any conduct that causes physical, psychological, or sexual damage within the relationship. Women's Abuse Screening Test was used; the rating level ranges 0–16 where a rating > 1 within a year suggests the presence of violence using by *Coker, Ann L; Pope, Brian O, *et al. [27].

#### Life events

Discrete stories that disrupt an individual's regular activities. List of threatening experiences (LTE) are a self-pronounced questionnaires which have 12 threatening activities that invite respondents to response question (yes/no) validated by *Brugha Ts and Cragg D*. threatening activities within the past 6-months become used. demonstrated good test–retest reliability of 89%. Participants with an LTE score of ‘0’ had been categorized as “No” and labeled as “0” and people who scored “1–12” had been categorized as. “Yes” and categorized as “1” [28].

#### A household food security

The ability of the household to secure adequate food intake for meeting the dietary need of all family members. A Household food security become assessed the use of the Household Food Insecurity Access Scale (HFIAS), for the use of program of the U.S. Agency for international development by the Food and Nutrition Technical Assistance (FANTA), which is verified by Salarkia, N., Abdollahi, M., Amini, M. et al*.* It has 9 questions with consecutive terms which is related to the regularity of incidence, if individual have a response is “yes” for the questions. A HFIAS rating levels a range from 0 to 27. In this study, household food security status categorized into two as “food secure” when the individual didn’t experience food inaccessibility conditions in the past 4 weeks and “food insecure” when individuals had unable to access enough food for necessary day to day activities [29, 30].

#### Depression

Depression was assessed using DASS-21 depression subscale which was; developed by Lovibond Sh, individuals who scored greater than or equal to 21 was considered as having depression [31]. Overall, the findings suggest that the DASS-21 is a useful self-report screening tool for depression, anxiety, and stress for screening.

#### Anxiety

Anxiety was assessed using DASS-21 anxiety subscale, which was; developed by Lovibond Sh, individuals who scored greater than or equal to 15 was considered as having anxiety [31].

#### Stress

Stress was assessed using DASS-21 stress subscale, which was; developed by Lovibond Sh, individuals who scored greater than or equal to 26 was considered as having stress [31].

#### Perceived social support

Support at time when difficulties and critical conditions like financial, social, and psychological, assessed by Oslo-3 social support scales which has total scores of 14 and classified into three broad categories by Kocalevent, RD., Berg, L., Beutel, M.E.2018. According to this respondents who will score 3–8 will be indication of as having poor social support, those who will score 9–11 will be indication of as having moderate social support and those who will score 12–14 will be indication of as having strong social support [32].

#### Harmful drinking

Fast Alcohol Screening Test (FAST) scale derived from the Alcohol Use Disorders Identification Test (AUDIT) by Hodgson et al., 2002.; was used to measure harmful drinking, FAST has 4 items with a rating score from a range of 0 to 16, where in a rating of > 3 shows harmful drinking [30, 33].

### Data analysis

First, data were entered into Epi-data version 3.1 then exported to SPSS-20 version for further analysis. Descriptive statistics was carried out for different variables to characterize the study population. Both bivariate and multivariate logistic regressions is used to identify associated factors. A variable that has p value ≤ 0.2 in the bivariate analysis was fitted into a multivariate logistic regression model to manage the impacts of confounding. Crude and adjusted odds ratio with 95% CI calculated to determine the strength and presence of association. P value of < 0.05 considered declaring the level of significance.

## Results

### Sociodemographic variables of respondents

Despite, our sample size is 845 only 804 responded to the interview with a response rate of 95%. Respondents had mean age of 32.0(SD ± 7.7) years. More than 80% (647) of reproductive age women were married and out of the total respondents 75.7% (609) living with their husbands. Three hundred seventy-six (46.8%) had no formal education, majority of women 80.2% (645) were rural residents. Almost 99.3% (798) of women orthodox Christian followers. Even though they are reproductive age group women only 15.2% (122) of are pregnant during time of data collection. Regarding their occupational status 32% (257) were housewife and one hundred thirty-nine (17.3%) have no children and 45.4% (365) had food insecurity problem (Table 1).

**Table 1:** Socio-demographic characteristics of women at reproductive age women in central Gondar zone, Northwest Ethiopia, 2021(n = 804)

Variables	Frequency	Percent
Age		
15–30	370	46
≥31	434	54
Marital status		
Non married	157	19.5
Married	647	80.5
Educational status		
No formal education	376	46.8
Primary education	208	25.9
High school and above	220	27.4
Living status		
Living with husband	609	75.7
Living with other relatives	195	24.3
Residence		
Rural	645	80.2
Urban	159	19.8
Religion		
Orthodox	798	99.3
^a^Other religion	6	0.7
Current pregnancy		
No pregnancy	682	84.8
Pregnant	122	15.2
Ethnicity		
Amhara	777	96.6
^a^nother ethnic group	27	3.4
Occupation		
^c^Other occupation	547	68
House wife	257	32
Number of children		
No children	139	17.3
1–3 Children	339	42.2
> 3 Children	326	40.5
Household food security		
Food secure	439	54.6
Food insecure	365	45.4

^a^Muslim, Protestant

^b^Tigre, Qumant

^c^Farmer, merchant, student

#### Prevalence of partner violence

The prevalence of intimate partner violence was 48.6% (391). Among the respondents 35.6% (286) had psychologic violence**,** A 23.4% (188) violated physically and two hundred twenty-five (28%) of women were encounter sexual violence. By considering psychosocial problems that contribute to intimate partner violence, 48.6% (391) had exposure to stressful life events,11.9% (96) had poor social support and 14.4% (116) engage in hazardous alcohol use behavior (Table 2).

**Table 2:** Distribution of psychosocial characteristics of reproductive age women in central Gondar zone, Northwest Ethiopia, 2021

Variable	Frequency	Percent
Exposure to stressful life events		
Yes	391	48.6
No	413	51.4
Social support		
Poor	96	11.9
Moderate	355	44.2
Strong	353	43.9
Hazardous alcohol abuse		
Yes	116	14.4
No	688	85.6

#### Clinical factors

Sixty-eight women (8.5%) had depression, eighty-two (10.2%) had anxiety, sixteen (2%) had stress, twenty-five individuals (3.1%) had history of mental illness once in life (Table 3).

**Table 3:** Distribution of clinical problem of reproductive age women in central Gondar zone, Northwest Ethiopia, 2021

Variable	Frequency	Percent
Depression		
Yes	68	8.5
No	736	91.5
Anxiety		
Yes	82	10.2
No	722	89.8
Stress		
Yes	16	2
No	788	98
History of mental illness		
Yes	25	3.1
No	779	96.9
Gynecological problem		
Yes	38	4.7
No	766	95.3
History of Abortion		
Yes	121	15
No	683	85

### Determinants of intimate partner violence

After adjustment seven variables were significantly associated with intimate partner violence: marital status, educational status, number of children, household food insecurity, social support, having stressful life events, having depression. Those reproductive age women who are married were 3.85(AOR = 3.85, 95% CI 2.38, 6.22) times more likely to have partner violence among currently non married individuals. The likely hood of partner violence was 57% (AOR = 0.43, 95% CI 0.30, 0.61) lower among women with high school and above education as compared to no formal education and again women with moderate social support were 40% (AOR = 0.60, 95% CI 0.41, 0.83) decreased among women with strong social support. Intimate partner violence was 1.82 times high (AOR = 1.82, 95% CI 1.0, 3.1) among participants having greater than three children as compared to participants with no children. Having household food insecurity increases the odds of intimate partner violence by 2.1 times (AOR = 2.09, 95% CI 1.51, 2.91) when compared to participants who had no food insecurity. Odds of exposed to intimate partner violence were 2.97 times (AOR = 2.97, 95% CI 2.11, 4.17) increment among participants who got life-threatening events in the past six months. Depression has higher odds of intimate partner violence by three times (AOR = 3.12, 95% CI 1.60, 6.07) as to participants who were free from depression (Table 4).

**Table 4:** Multivariate Logistic regression on intimate partner violence among reproductive age women in central Gondar, Northwest Ethiopia,2021

Characteristics	IVP		COR	AOR	P-value
	No	Yes			
Age of participants					
15–30	214	156	1	1	
≥31	199	235	1.6 (1.2,2.14) *	1.01 (0.69,1.50)	0.91
Marital status					
^a^Nonmarried	119	38	1	1	
Married	294	353	3.76 (2.52,5.6) *	3.85 (2.38,6.22) **	0.001
Residence					
Rural	315	330	1	1	
Urban	98	61	1.68 (1.18,2.4) *	1.14 (0.64,1.94)	0.7
Education					
No formal education	172	204	1	1	
Primary Education	96	112	2.3 (1.62,3.23) *	0.98 (0.70,1.38)	0.92
High school & above	145	75	2.25 (1.52,3.33) *	0.43 (0.30,0.61) **	0.001
Occupation					
^b^Other occupation	254	293	1	1	
House wife	159	98	1.9 (1.38,2.53) *	0.72 (0.46,1.13)	0.15
Living condition					
With husband	276	333	2.85 (2.01,4.02) *	1.3 (0.77,2.16)	
With other family	137	58	1	1	
Pregnancy status					
Not pregnant	361	321	1	1	
Pregnant	52	70	1.51 (1.02,2.23) *	1.21 (0.77,1.91)	0.41
History of abortion					
Yes	47	74	1.8 (1.22,2.70) *	1.10 (0.69,1.75)	0.67
No	366	317	1	1	
Number of children					
No children	102	37	1	1	
1–3 Children	176	162	2.5 (1.66,3.93) *	1.53 (0.93,2.51)	0.09
> 3 Children	135	191	3.9 (2.52,6.00) *	1.82 (1.0,3.1) **	0.02
Household food security					
Food secure	263	176	1	1	
Food insecure	150	215	2.14 (1.61,2.84) *	2.09 (1.51,2.91) **	0.001
Social support					
Poor	52	44	0.71 (0.44,1.10)	0.64 (0.37,1.09)	0.1
Moderate	201	154	0.63 (0.47,0.85) *	0.60 (0.41,0.83) **	0.003
Strong	160	193	1	1	
Life events					
No	262	151	1	1	
Yes	151	240	2.76 (2.07,3.67) *	2.97 (2.11,4.17) **	0.001
Harm full drinking					
No	366	322	1	1	
Yes	47	69	1.67 (1.12,2.49) *	1.35 (0.82,2.20)	0.22
Depression					
No	392	344	1	1	
Yes	21	47	2.56 (1.50,4.35) *	3.12 (1.60,6.07) **	0.001
Anxiety					
No	387	335	1	1	
Yes	26	56	2.49 (1.53,4.05) *	1.66 (0.82,3.36)	0.15
Stress					
No	410	378	1	1	
Yes	03	13	4.7 (1.33,16.62)	2.10 (0.46,9.48)	0.33

^a^Single, divorced, widowed, separated

^b^Farmer, merchant, student

## Discussion

In this community-based study near to half 48.6% (45, 52) of women had intimate partner violence. Being married, increased number of children, having household food insecurity, presence of stressful life events, depressive symptoms increase the chance of intimate partner violence. Our finding is similar with studies conducted in arbaminch Ethiopia 50% [34], eastern Ethiopia 45.7% [35], in Jimma (45%) [36]. On the contrary, this study is higher than the study conducted in Ethiopia EDHS 30% [37], urban Ethiopia 32% [38], married women in Ethiopia 22.4% [39], systematic review in Ethiopia 44% [40], Uganda36.6% [41]. Our finding is decreased in prevalence than the study conducted in wolaita Ethiopia 59.7% [42], Dadaab refugee kenya 66.7% [43], low and middle income countries 55% [44], Uganda married women 56% [45]. These discrepancies are due to differences socio-economic status and differences in research methods, the study participants of our study focus on reproductive age women, but other on pregnant women.

Those reproductive age women who are married were 3.85 times more likely to have partner violence among currently not married individuals. This finding is not on the same track with the study conducted in South Africa [46], the Demographic and Health Survey in sub-Saharan African countries [47], pregnant women in Ethiopia [48]. The reason may be married ones have long duration contact each other to live together and more likely to compromise on certain issues which brings high conflict in their homes. Another possible reason might be due to the sociocultural value in the communities with male dominancy.

The likely hood of partner violence was 57% decreased among women with high school and above education as compared to women with no formal education. This result is the same with the study done in India wives with higher education were decreased to experience violence [49], South Africa women with no education, primary education and secondary education were vulnerable to experience IPV compared to those with higher education [47], Gahana senior high school education or higher was protective of IPV [50].The possible justification is that women’s educational status is more linked with increased chance of getting paid jobs, increased odds of status gain and a better balance of power in marriage [51, 52], increasing women’s schooling also reduced their probability of experiencing any form of IPV at all and their probability of experiencing two or more forms of IPV relative to none [53].

The study also demonstrated that the odds of developing partner violence among women that have moderate social support were 40% lower among women with strong social support. Thus, consistent with previous study done on six European countries social support may have positive effect to decrease violence [54], Tanzania social support shows decreased odds of IPV and repeated episodes of IPV [55]. The main reason is that getting social support from friends and family members linked to less victimization among women, not having social support would increase partner violence.

Our study also revealed that having greater than three children increase the odds of partner violence as compared to participants with no children by two-fold. No previous study supports our finding.

This finding has revealed odds of IPV among women who had household food insecurity is high than women who had no food security problem. This finding supported by previous published study where women in African-American had a higher prevalence of food insecurity and were added report severe intimate partner violence [56], in Uganda food insecurity increase risk of both physical and sexual violence [57], South Africa, food insecurity double the odds of intimate partner violence [58], USA food insecurity associated with violence [59], California USA, women who have high prevalence of food insecurity were more prone to report severe intimate partner violence [56]. The possible justification is that food insecurity causes poor nutritional status [60], that contribute to develop mental disorder such as depression [61], Which may also make a contribution to accelerated marital misery and violence [62, 63], and due to many macro-structural reasons including women considered as homemade and culturally dominated by men this causes power inequality that cause greater risk of experiencing food insecurity.

This study also shows that strong association of life-threatening events and partner violence. Women that encounter life-threatening events in the previous six months were almost three more odds of intimate partner violence than those women who did not encountered life-threatening events. But we did not get any finding that supports our findings.

Those reproductive age women with depression more experienced intimate partner violence three times higher compared to women without depression. Our finding is supported by different study conducted in Gahana [50], Nigeria [64]. On the other hand intimate partner violence increase prevalence depression among women in reproductive age women like community based study conducted in Ethiopia [65], Bangladesh [66], South Africa [67], Australia [68]. Studies show that intimate partner violence (IPV) is increased risk of psychological distress, in addition patients with mental health problem are double burden for violence and it is difficult to say one has casual relation with the other.

## Conclusions

In the research area, nearly half of women of reproductive age experienced intimate partner violence. Intimate partner violence was linked to being married, having number of children, experiencing life-threatening events, household food insecurity, a lack of social support, a lack of or a low level of education, and having depressive symptoms. However, characteristics including as pregnancy, abortion history, husband educational status, and husband history of substance use, which have a strong correlation with violence, had no association in our findings. A future qualitative longitudinal study could investigate the relationship between intimate partner violence and determining factors.

### Limitations

Because of its cross-sectional character, this finding may not establish cause and effect relationships between IPV and independent variable. Furthermore, recall bias and social desirability must be considered; culturally, some of the features of the measuring tool may not be violating women's rights. Despite its faults, the current study provides evidence and useful data on the prevalence of intimate partner violence among women of reproductive age.

## Data Availability

The datasets used and/or analyzed during the current study available from the corresponding author on reasonable request at wondale22@gmail.com/Wondale.getinet@uog.edu.et. It is ongoing mega project and request from the university is recommended with www.uog.edu.et.
